# Antibacterial activity of silver bionanocomposites synthesized by chemical reduction route

**DOI:** 10.1186/1752-153X-6-101

**Published:** 2012-09-12

**Authors:** Mansor Bin Ahmad, Jenn Jye Lim, Kamyar Shameli, Nor Azowa Ibrahim, Mei Yen Tay, Buong Woei Chieng

**Affiliations:** 1 Department of Chemistry, Faculty of Science, University Putra Malaysia, Selangor, Malaysia; 2 Materials & Energy Research Center, P.O. Box: 31787/316, Alborz, Karaj, Iran

## Abstract

**Background:**

The aim of this study is to investigate the functions of polymers and size of nanoparticles on the antibacterial activity of silver bionanocomposites (Ag BNCs). In this research, silver nanoparticles (Ag NPs) were incorporated into biodegradable polymers that are chitosan, gelatin and both polymers via chemical reduction method in solvent in order to produce Ag BNCs. Silver nitrate and sodium borohydride were employed as a metal precursor and reducing agent respectively. On the other hand, chitosan and gelatin were added as a polymeric matrix and stabilizer. The antibacterial activity of different sizes of silver nanoparticles was investigated against Gram-positive and Gram-negative bacteria by the disk diffusion method using Mueller-Hinton Agar.

**Results:**

The properties of Ag BNCs were studied as a function of the polymer weight ratio in relation to the use of chitosan and gelatin. The morphology of the Ag BNCs films and the distribution of the Ag NPs were also characterized. The diameters of the Ag NPs were measured and their size is less than 20 nm. The antibacterial trait of silver/chitosan/gelatin bionanocomposites was investigated. The silver ions released from the Ag BNCs and their antibacterial activities were scrutinized. The antibacterial activities of the Ag BNC films were examined against Gram-negative bacteria (*E. coli* and *P. aeruginosa*) and Gram-positive (*S. aureus* and *M. luteus*) by diffusion method using Muller-Hinton agar.

**Conclusions:**

The antibacterial activity of Ag NPs with size less than 20 nm was demonstrated and showed positive results against Gram-negative and Gram-positive bacteria. The Ag NPs stabilized well in the polymers matrix.

## Background

Over last few decades, nanomaterials exhibit unique, superior and very important properties. Due to their distinct characteristics, attention was drawn to represent nanomaterial into a significant group of materials in the development of novel devices which can be used in various biological, chemical, physical, biomedical and pharmaceutical applications. Reports have shown promising results about the activity of different drugs and antimicrobial formulation in the form of nanoparticles. Silver nanoparticles (Ag NPs) have attracted intensive research interest for centuries because of their important biological applications especially in bactericidal effect which is the capability of killing about 650 types of diseases causing microorganisms [1]. It has a significant potential for preventing infections, healing wounds [2] and anti-inflammatory. Hence, Ag NPs had been incorporated in textile fabrics, polymers, dental material, medical device and burn dressing to eliminate microorganisms.

During the synthesis of Ag NPs, stabilizers play a main role in controlling the size of particles as well as their dispersion stability. Polymers, often used as stabilizers due to the effectiveness in preventing agglomeration and precipitation of the particles provide to excellent distribution of particles. A lot of researches have been discovered that various polymer materials, such as polyethylene glycol, [3] poly (vinyl alcohol), [4] poly (lactic acid), [5] polyacrylonitrile, [6] chitosan, [7,8] poly (N-vinylpyrrolidone), [9] and gelatin, [10-12] could be used as polymer matrices. There are a variety of ways to consolidate Ag NPs into polymer matrix which included chemical reducing, [6,13] radiochemical, [14,15] photochemical, [16] electrochemical, [10,11] and sonochemical [17] method.

Gelatin, a heterogeneous mixture of high molecular weight water-soluble proteins derived from partial hydrolysis of naturally occurring collagen, contains relatively high amounts of non-polar amino acids such as Gly, Pro, Val and Ala [18]. From years of research, fish skin waste was reported as possible potential source for collagen isolation. Collagen structure is a triple helix which consists three extended protein chains that wrap around each other and gelatin is the partially hydrolyzed form of collagen. The repetitive Gly-Pro-Ala sequence in gelatin peptides’ structure was reported to the antioxidative, antihypertensive as well as increasing calcium bioavailability is the reason of its wide research in bone engineering field [19]. Furthermore, unique properties of gelatin which are nontoxicity, biodegradable, biocompatible, and nonimmunogenic abilities lead itself capable for biomedical applications, for example in drug delivery as capsules, hydrogel, or microspheres [20].

Chitosan(Cts), a polysaccharide biopolymer derived from naturally occurring chitin, is an excellent natural polymer due to its nontoxicity, biodegradability, biocompatibility, bioactivity, multifunctional groups and antimicrobial activity. The shell wastes are presumed as potential sources to isolate chitin and chitosan. Both chitin and chitosan are linked through *β*-d-1, 4-glycosidic bonds units [18]. According to the chemical structure, the existence of active amino and hydroxyl functional groups in chitosan lead to various properties such as unique cationic, chelate metal ions, ability to form films, and optical structural character [21]. As a result, it is extensively being investigated in field of agriculture, food packaging industry, bone engineering, artificial skin, biomedical material and drug delivery [22].

Polymer blending is one of the most efficient methods promising for novel engineered as well as desirable composite materials for numerous potential applications [23] yet the important highlight of the features of a composite is the compatibility of its components. Compatibility between chitosan and gelatin is dependable to specific interactions between polymeric components which are the ability of carboxyl groups of gelatin to form hydrogen bonding with chitosan. [24] In term of biological activity, chitosan/gelatin composite had achieved improvement compared to its components since gelatin able to promote cell adhesion and migration as well as forms a polyelectrolyte complex. Many studies had been carried out on the synthesis of chitosan/ gelatin composite. Chitosan-gelatin scaffolds have been formed without or with cross-linkers such as genipin, [25] glutaraldehyde or enzymes [26] and studied in various tissues regeneration [27].

The antibacterial effect of Ag NPs in chitosan and gelatin respectively were reported [8,11]. However, reports on the effect of antibacterial activity of Ag NPs in chitosan/gelatin which has potential application in biomedical field had not been found.In this research, Ag NPs had been synthesized by chemical reduction method within the suspension of two biopolymers, i.e., chitosan and gelatin. The reducing agent used in this study is sodium borohydride. The final product of Ag NPs in polymers is labeled as Ag BNCs. Characterization of the prepared Ag BNCs have been investigated on their surface plasmon resonance bands, morphology, mean diameter of nanoparticles, nanoparticles’ distribution and crystalline structures. Next, the antibacterial activities of the BNCs toward Gram-positive and Gram-negative bacteria are studied.

## Results and discussion

### Characterization

In this research, Cts, Cts/gelatin and gelatin suspension with different weight ratio are employed as stabilizers for the Ag NPs which formed by chemical reduction process. Sodium borohydride which is a strong reducing agent was successfully reduced AgNO_3_ in the suspension of Cts, gelatin as well as combination of both polymers, and lead to the formation of Ag NPs.

**Figure 1:**
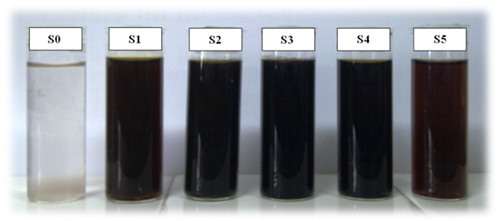
**Picture of AgNO**_**3**_**/Cts/gelatin (S0), Ag/Cts (S1), Ag/Cts/gelatin (S2-S4) and Ag/gelatin.**

**Figure 2:**
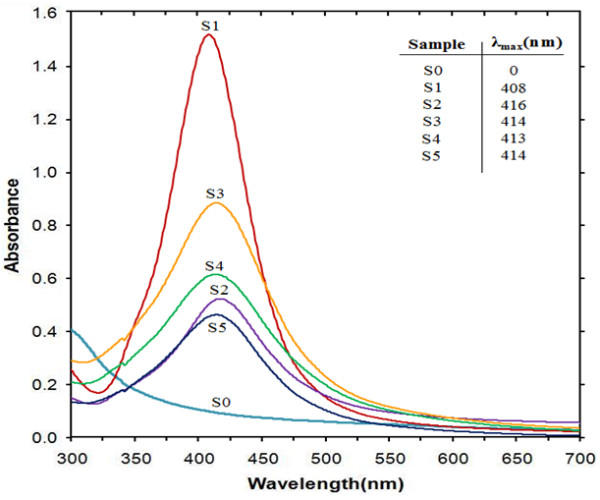
**Ultraviolet–visible absorption spectra of AgNO**_**3**_**/Cts/gelatin (S0), Ag/Cts (S1), Ag/Cts/gelatin (S2- S4), and Ag/gelatin (S5) BNCs suspension.**

**Figure 3:**
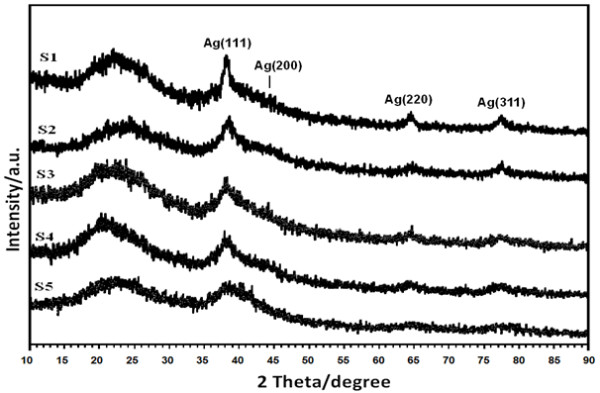
Powder X-ray diffraction patterns of Ag/Cts (S1), Ag/Cts/gelatin (S2-S4) and Ag/gelatin (S5) BNCs for Ag crystals structure determination.

AgNO_3_/Cts/gelatin BNCs (S0) was colorless, but once the reducing agent was introduced to the polymers suspensions (S1-S5), the immediate color changed indicating the formation of Ag NPs in the Cts, Cts/gelatin and gelatin. Samples labeled S1 and S5 were brown, whereas S2, S3 and S4 were dark brown as shown in Figure 1. Surface plasmon resonance bands of all the suspensions (S0-S5) are presented in Figure 2, measured at wavelength from 300 to 700 nm to confirm the existence of Ag NPs in suspensions (S1-S5). For detection of XRD pattern, the suspensions were prepared into thin films. The XRD patterns for S1-S5 were detected in the wide angle range of 2θ (10° < 2θ < 90°) for the determination of Ag NPs’ crystalline structures. (Figure 3)

**Figure 4:**
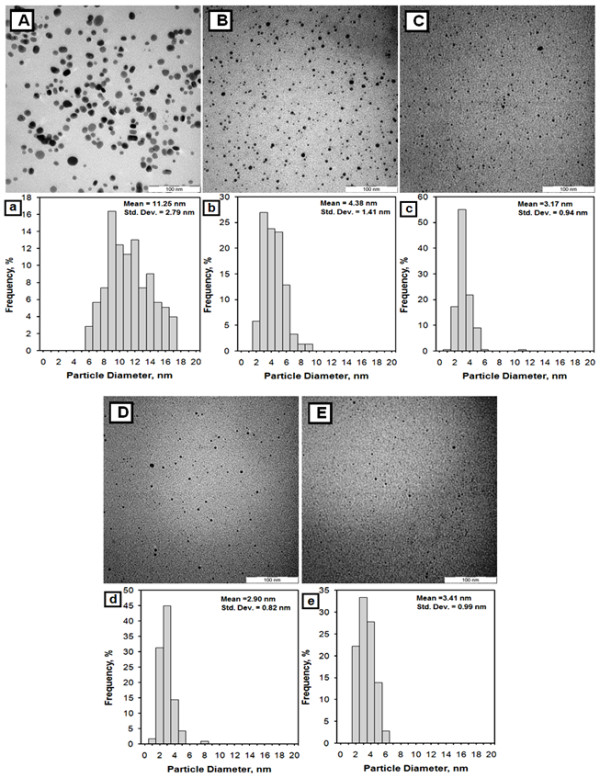
Transmission electron microscopy images and corresponding particle size distribution histograms for S1 (A,a), S2 (B,b), S3(C,c), S4(D,d) and S5(E,e) BNCs suspension.

**Figure 5:**
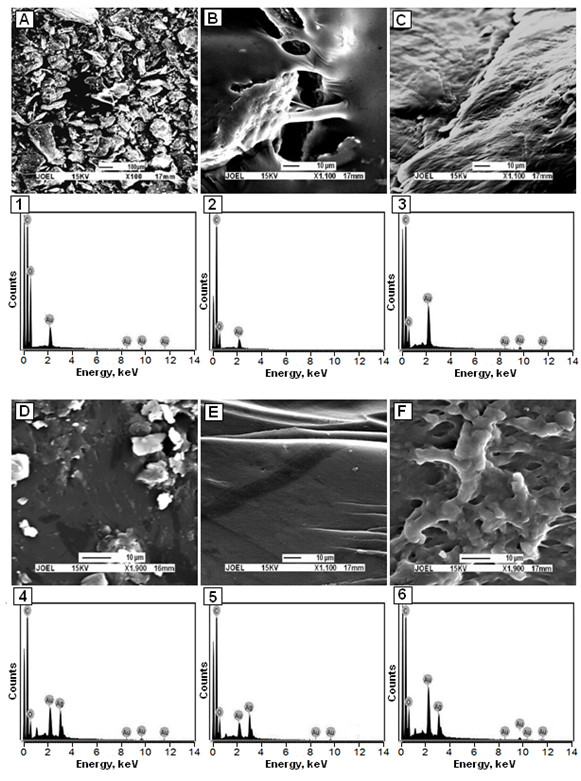
SEM-EDX images of the surface morphology of Cts (A,1), Cts/gelatin (B,2), gelatin (C,3), Ag/Cts BNCs-S1(D,4), Ag/Cts/gelatin BNCs-S3 (E,5) and Ag/gelatin BNCs-S5(F,6).

**Figure 6:**
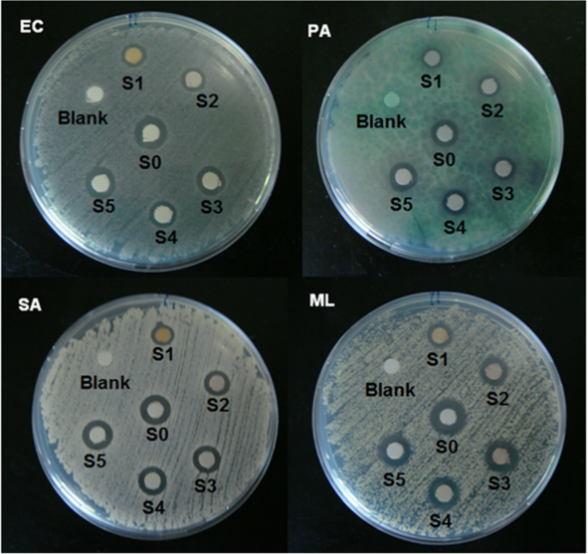
**Comparison of the inhibition zone test between Gram-negative bacteria (*E. coli *and *P. aeruginosa *) and Gram-positive (*S. aureus *and *M. luteus*) for AgNO **_**3**_**/Cts/gelatin (S0), Ag BNCs (S1-S5) and Cts/gelatin (Blank).**

The TEM images of Ag BNCs and the histogram in Figure 4 illustrate the Ag particles distributions in suspension with average mean diameter of the nanoparticles less than 20 nm. The SEM images with EDX detection spectra are shown in Figure 5. The SEM images highlighted the additional information for the surface morphology of the pure polymers and Ag BNCs. In addition, the EDX spectra for the Cts, Cts/gelatin, gelatin and Ag BNCs (S1, S3, and S5) confirmed the presence of elemental compounds in Cts, gelatin and Ag NPs without any other impurity peaks. The antibacterial studies had showed relatively similar effects for all samples indicated by the inhibition zone test between Cts/gelatin, AgNO_3_/Cts/gelatin (S0), and Ag BNCs (S1-S5) against different bacteria (Figure 6, Table 1).

**Table 1:** **Average diameter of inhibition zone for Cts/gelatin, AgNO**_**3**_**/Cts/gelatin, Ag/Cts (S1), Ag/Cts/gelatin (S2-S4) and Ag/gelatin (S5)**

**Diameter of inhibition zone (mm)**							
**Bacteria**	**Samples**					**Control negative**	** Control positive**
	**S1**	**S2**	**S3**	**S4**	**S5**	**Cts/gelatin (Blank)**	**AgNO**_**3**_**/Cts/gelatin(S0)**
*E. coli*	8.8	9.0	9.9	9.2	10.3	Not Appearing	10.9
*P. aeruginosa*	8.2	9.0	8.7	9.0	10.1	Not Appearing	10.6
*S. aureus*	8.8	9.6	9.9	9.6	10.4	Not Appearing	11.1
*M. luteus*	9.2	10.6	11.4	11.0	11.6	Not Appearing	12.9

During the NaBH_4_ reducing process, color of the AgNO_3_/Cts/gelatin suspensions changed from colorless to brown. The color changes due to the formation of Ag NPs are proven by UV-visible spectra in Figure 2. There is no surface plasmon resonance band observed in between 300–700 nm range for AgNO_3_/Cts/gelatin (S0) indicated that there is no Ag NPs were formed before the addition of NaBH_4_. After adding NaBH_4_, the maximum absorbance bands for S1, S2, S3, S4 and S5 were detected at 408, 416, 414, 413, and 414 nm respectively. These absorption bands were indication of Ag NPs formation with small diameter size [28]. The diameter sizes of the Ag NPs formed were further established with the data from TEM, which reveal the diameter of the Ag NPs. The absorption peak of Ag for S1 (408 nm) was in the blue-shifted state compared to S2 (416 nm), S3 (414 nm), S4 (413 nm), S5 (414 nm). The SPR absorption band and TEM results showed signs of an exceptional case: The particle size in S1 with mean diameter 11.25 nm have absorbance at blue-shifted wavelength compared to other samples that the average diameter is less than 5 nm. The cause of the red-shift is due to the lowered conductivity in the outer Ag metallic layer resulted by chemical interactions between Ag NPs, Cts, and gelatin. This phenomenon was well explained by Mie theory [29].

In Figure 3, the Ag BNCs (S1-S5) had similar x-ray diffraction patterns at 2θ with value of around 38°, 44°, 64°, and 77° which corresponding to the 111, 200, 220, and 311 crystallographic planes of the face-centered cubic Ag crystals, respectively. (JCPDS file No. 00-004-0783) [30]. The broad peaks observed from 10° to 35° contribute to the structure of the Cts and gelatin. These XRD patterns further confirmed the result of UV spectra that is the existing of Ag NPs in the Cts, Cts/gelatin and gelatin as final product.

The TEM images of particles and their diameter sizes distribution histograms for Ag BNCs (S1-S5) are shown in Figure 4. Mean diameters of Ag NPs revealed were 11.25 ± 2.79, 4.38 ± 1.41, 3.17 ± 0.94, 2.90 ± 0.82, and 3.41 ± 0.99 nm for Ag/Cts (Figure 4 A, a), Ag/Cts/gelatin (Figure 4 B-D, b-d) and Ag/gelatin (Figure 4 E, e), respectively. The TEM results indicate that the Ag NPs formed in Cts, Cts/gelatin and gelatin suspension retained a narrow particle size distribution and the least diameters of the Ag NPs are achieved in S4 with weight ratio of Cts/Gelatin = 0.7 g/0.3 g. TEM image of Ag/Cts also showed some aggregation of Ag NPs whereas TEM image of Ag/gelatin showed fewer occurrences of Ag NPs within the gelatin suspension. With the combination of Cts and gelatin, their TEM image had illustrate good distribution and higher occurrence of the Ag NPs.

Surface morphology of polymer and Ag BNCs are illustrated in Figure 5. The samples for SEM analysis were prepared by solvent casting on petri dish. The pure Cts and gelatin was showed in the Figure 5A and C respectively. The blending of Cts and gelatin in film form showed some smooth surface and pores as shown in Figure 5B. The SEM images for Ag BNC are shown in Figure 5 D-F. From the SEM image, Ag/Cts (S1) BNCs showed show layered surfaces with small flakes, Ag/Cts/gelatin (S3) showed smooth layered surfaces and Ag/gelatin (S5) showed porous layered surfaces. Effect of Ag NPs in S3 was clearly shown in the SEM images where Cts/gelatin composite has pores after solvent casting whereas Ag/Cts/gelatin remained in smooth surfaces. In addition, the EDX spectra for the Cts, Cts/gelatin, gelatin and Ag BNCs (S1, S3, and S5) had confirmed the presence of elemental compounds in the Cts, gelatin and Ag NPs without any impurity peaks. All the samples tested for EDX were coated with gold to prevent the accumulation of static electric fields during imaging. The Ag BNCs film morphologies were dependent on several factors including polymer solubility, solvent evaporation, total thickness, molecular weight and surface composition [31].

### Antibacterial study

Inhibition zone values were obtained from the synthesized Ag NPs tested against Gram-negative bacteria (*E. coli* and *P. aeruginosa*) and Gram-positive (*S. aureus* and *M. luteus*). Cts/gelatin was use as control negative as there is no antibacterial effect found while the AgNO_3_/Cts/gelatin as control positive due to its high antibacterial outcome. Figure 6 illustrate the images of each inhibition zones for the samples for antibacterial activity studies. Results of the inhibition zones are presented as average values in mm in the Table 1. The table shows that the Ag NPs had high and similar antibacterial activity against Gram-positive and Gram-negative bacteria. Due to their particle size, Ag NPs can easily reach the nuclear content of bacteria by disrupt the membranes of bacteria. The particle size smaller than 10 nm interact with bacteria and generate electronic effects that improve the reactivity of Ag NPs [32]. The antibacterial activity of Ag BNCs on *E. coli, S. aureus* and *M. luteus* have the same pattern that is Ag/gelatin (S5) have the highest antibacterial activity followed by Ag/Cts/gelatin S3 then S4, S2 and finally Ag/Cts S1. Ag/gelatin (S5) has the strongest antibacterial action on *P. aeruginosa* followed by S2 and S4, then S3 and finally S1. From Table 1, the results show a common pattern that is Ag/Cts which having average diameter of 11.25 nm have the least inhibition diameter due to their particle sizes is bigger than other samples. Ag/gelatin exhibit the largest bacteria inhibition zone among the samples might due to the weak stabilizing ability and its water solubility, hence lead to the ease of releasing Ag nanoparticles into the agar plate.

## Conclusions

A simple way to prepare Ag NPs in chitosan and gelatin by reacting AgNO_3_ in chitosan, gelatin and both with sodium borohydride as reducing agent was developed. The average diameters of the Ag NPs were between 2.90-11.25 nm with well crystallized structures. The XRD pattern confirmed the crystallographic planes of the Ag crystal are the fcc types. UV–vis absorption spectra show peaks characteristic of the surface plasmon resonance of Ag NPs. The antibacterial activity of Ag BNCs was demonstrated and showed antibacterial activity against Gram-negative and Gram-positive bacteria.

## Methods

### Materials

All reagents in this research were analytic grade and used as received without further purification. AgNO_3_ (99.98%), as the silver precursor, was obtained from Merck (Darmstadt, Germany). The sodium borohydride (NaBH_4_), the reducing agent, was obtained from Sigma–Aldrich (St. Louis, MO, USA). Chitosan with low molecular weight and glacial acetic acid (99%) were also obtained from Sigma–Aldrich whereas gelatin was purchased from HiMedia (Bombay, India). All the aqueous solutions were prepared with double-distilled water.

### Synthesis of Ag/Chitosan BNCs

The chitosan suspension was prepared by solubilizing 1.0 g of chitosan in 50 mL HAC (1.0 wt%) solution at room temperature. Then, 50 mL of AgNO_3_ (0.01 M) was added immediately into the suspension under constant stirring for 2.0 hours for preparation of the AgNO_3_ in chitosan suspension. The 20 mL of NaBH_4_ (0.04 M) was added to the suspension of AgNO_3_/Cts and the immediate color changes from pale yellow to brown indicating the formation of Ag NPs. This suspension was continued stirring for 1.0 hour. Then, the obtained Ag/Cts BNCs were labeled as S1 and further characterization was carried out.

### Synthesis of Ag/Chitosan/Gelatin BNCs

Different weight of chitosan was dissolved in 25 mL acetic acid (1.0 wt%) solution. On the other hand, different weight of gelatin was dissolved in 25 mL of warm distilled water (40°C). The weight ratio of chitosan and gelatin for sample S2, S3 and S4 is shown in Table 2. Both suspensions were mixed, and 50 mL of AgNO_3_ (0.01 M) was added directly into the suspensions. The AgNO_3_/Cts/Gelatin suspension was stirred for 2.0 hours. The 20 mL of NaBH_4_ (0.04 M) was added immediately to the suspension of AgNO_3_/Cts/gelatin. A color change from colorless to dark brown was observed. The suspension was stirred for another 1.0 hour. Then, the obtained Ag/Cts/gelatin BNCs were ready for characterization.

**Table 2:** Weight ratio of Cts and gelatin for Ag BNCs with label

**Label**	**Cts/Gelatin Ratio (g/g)**
S2	0.7 / 0.3
S3	0.5 / 0.5
S4	0.3 / 0.7

### Synthesis Ag/Gelatin BNCs

For gelatin suspension preparation, 1.0 g of gelatin was solubilized into 50 mL of warm distilled water (40°C). Next, 50 mL of AgNO_3_ (0.01 M) was mixed into the suspension and stirring for 2.0 hours. Then, 20 mL of NaBH_4_ (0.04 M) was added immediately to the suspension of AgNO_3_/gelatin. Meantime, the immediate color change from colorless to brown was observed. This suspension was stirred for another 1.0 hour. The product was labeled as S5 and characterized.

### Characterization methods and instruments

The prepared Ag BNCs were characterized by UV-visible spectroscopy (UV-visible), X-ray diffraction (XRD), transmission electron microscopy (TEM), and scanning electron microscope with energy dispersive X-ray analyzer (SEM-EDX). The UV-visible spectra were detected over the range of 300–700 nm using Shimadzu H.UV.1650 PC UV-visible spectrophotometer. Crystalline structures of the synthesized Ag were examined using Philips X’pert Pro Panalytical PW3040MPD X-ray diffraction. TEM image observations were carried out on Hitachi H–7100 electron microscope and the particle size distributions were determined using the UTHSCSA Image Tool program (V. 3.00; University of Texas Health Science Center, San Antonia, TX). The SEM-EDX images of samples were obtained from JEOL Scanning Microscope JSM-6400.

### Evolution of antibacterial activity

The in vitro antibacterial activity of the samples was evaluated using the Mueller-Hinton Agar disc diffusion method with determination of diameter of inhibition zones in mm. *Escherichia coli* (ATCC 25922)*, Staphylococcus aureus* (ATCC 25923), *Micrococcus luteus*(ATCC 15307) and *Pseudomonas Afruginosa* (ATCC 27853) were used for the antibacterial effect assay. Briefly, sterile filter paper disc (6 mm) impregnated with 10 μL of Ag BNCs (S1-S5) with different weight ratio of cts and gelatin. The bacterial suspension was prepared by making a saline suspension of isolated colonies selected from 18 to 24 hours of tryptic soy agar plating. The suspension was adjusted to match the tube of 0.5 McFarland turbidity standard using spectrophotometry at 600 nm, which equals 1.5 × 108 colony-forming units/mL. The surface of the Mueller-Hinton Agar was completely inoculated using a sterile swab, which was steeped in the prepared suspension of bacteria. Finally, the impregnated discs were placed on the inoculated agar and incubated at 37°C for 24 hours. After incubation, the diameter of the growth inhibition zones was measured. AgNO_3_ (0.01 M) was used as the positive standards in order to control the sensitivity of the bacteria. All tests were done in duplicate.

## Competing interests

We declare that we have no competing interests.

## Authors’ contributions

JJL and MYT carried out the synthesis and characterization of the compounds. BWC carried out the antibacterial test for the material. MBA, NAI and KS conceived of the study and helped to draft the manuscript and problem solving. All authors read and approved the final manuscript.
